# Harnessing the Power of Microwave Irradiation: A Novel Approach to Bitumen Partial Upgrading

**DOI:** 10.3390/molecules28237769

**Published:** 2023-11-25

**Authors:** Moataz K. Abdrabou, Xue Han, Yimin Zeng, Ying Zheng

**Affiliations:** 1 Department of Chemical and Biochemical Engineering, Western University, London, ON N6A 3K7, Canada; mabdrab@uwo.ca; 2 Natural Resources Canada—CanmetMaterials, Hamilton, ON L8P 0A5, Canada; xue.han@nrcan-rncan.gc.ca (X.H.); yimin.zeng@nrcan-rncan.gc.ca (Y.Z.)

**Keywords:** microwave-assisted upgrading, bitumen partial upgrading, carbon susceptors, heavy oil processing, microwave energy

## Abstract

The partial upgrading of “tar-like” Canadian bitumen is an essential process to reduce its viscosity to an acceptable range that meets the required pipeline specifications. An innovative and potentially greener solution has emerged in the form of microwave irradiation. This work proposes and demonstrates the use of an electrically powered commercial microwave along with carbon-based microwave susceptors (activated carbon, biochar, coke, and graphite) to promote localized thermal cracking within bitumen at a temperature as low as 150 °C, compared to the conventional method of 400 °C. The remarkable results show that just 0.1 wt% of carbon additives can reduce the viscosity of bitumen by 96% with just 10 min of microwaving at 200 °C. A Saturates, Aromatics, Resins, and Asphaltenes (SARA) analysis reveals that the mass fractions of light components (saturates) are almost doubled and that almost one-third of heavy polar hydrocarbon constituents are cracked and decomposed into much lighter molecules, resulting in higher-quality, less viscous bitumen. Furthermore, this study highlights the key role of the surface area and porosity of the carbon microwave susceptor in absorbing microwave radiation, offering exciting new avenues for optimization. Microwave-assisted partial upgrading of bitumen is a cost-effective and eco-friendly alternative to conventional upgrading, producing upgraded bitumen that requires significantly less diluent at a lower cost prior to pipeline transportation.

## 1. Introduction

Bitumen, a highly viscous oil that represents over 50% of the current global oil reserves [1], presents a significant challenge for pipeline transportation due to its extremely high viscosity; density; and asphaltene content, which accounts for over 15% of its total weight [2]. Consequently, bitumen modification processes are needed to meet the pipeline specifications, which mandate a maximum viscosity of 350 cSt and a density of less than 940 kg/m^3^ at the reference temperatures [3]. Currently, the sole dilution of bitumen with expensive diluents such as condensate or light naphtha increases its production costs by approximately USD 14 per barrel of bitumen and occupies about 30–33% of the pipeline capacity [4]. Alternatively constructing new upgrading plants is no longer economically viable [5]. In addition to that, the current operating upgraders contribute significantly to greenhouse gas (GHG) emissions, with oil sand operations currently accounting for around 8% of Canada’s total GHG emissions [6]. As a result, some partial upgrading techniques that involve a combination of heating, diluent mixing, and upgrading to reduce viscosity by cracking macromolecules are alternatively being used [7]. Nonetheless, substantial viscosity reduction is typically achieved at relatively high temperatures of 400 °C + in processes such as visbreaking and/or coking [8], making traditional thermal upgrading techniques both energy-intensive and time-consuming [9] and ultimately leading to substantial GHG emissions. As such, the exploration of new innovative, green, and sustainable partial upgrading techniques is required.

**Figure 1:**
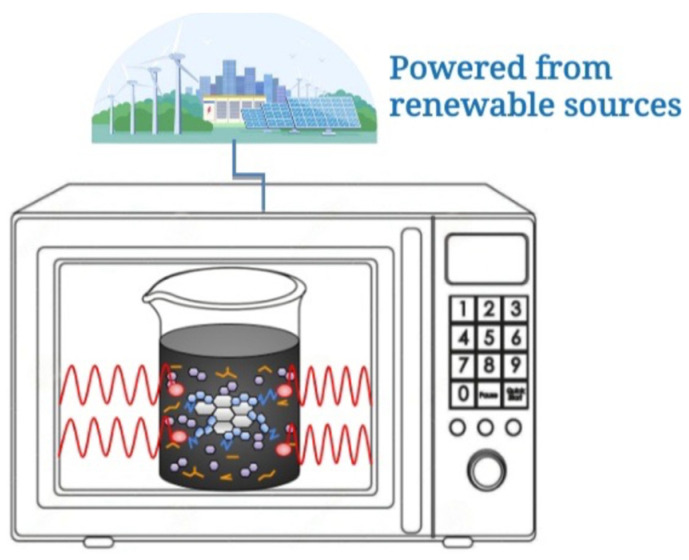
Schematic for the microwave heating of bitumen.

This paper introduces the concept of microwave irradiation as a promising alternative to conventional bitumen partial upgrading techniques. Unlike traditional heating methods, which heavily rely on natural gas combustion and have a high carbon footprint, microwave technology can be integrated with renewable energy sources, potentially reducing CO_2_ emissions to nearly zero. Despite higher operating costs currently preventing its adoption in refineries, microwave irradiation offers promising benefits, including selective and volumetric heating and a more convenient plug-on-and-plug-off mode for remote operation [10]. Figure 1 illustrates a proposed schematic for the upgrading process of bitumen via microwave irradiation.

Studies show that microwave heating can selectively target and crack highly polar hydrocarbon fractions within bitumen [11], greatly enhancing the quality of the upgraded oil. Evidence suggests that heavy oils rich in heteroatoms, like bitumen, can absorb more microwave energy, resulting in a significant reduction in viscosity under relatively mild conditions [11]. The ability of the oil to absorb and utilize the energy induced by microwave radiation greatly depends on two crucial parameters: the dielectric constant and the dielectric loss factor. The former parameter quantifies a material’s aptitude to store electromagnetic energy and undergo polarization, while the latter measures the conversion of this energy into heat [12]. Studies report that the dielectric constant of crude oil typically lies between 1.72 and 2.34 [10,11,12]. However, when examined in isolation, oil’s hydrocarbon fractions show distinct dielectric properties. Asphaltenes exhibit the highest dielectric constant values, ranging from 3.30 to 5.00 [10,11,12]. In contrast, the resin and aromatic fractions display lower dielectric constants, within the range of 1.80–2.61 and 2.0–2.7, respectively [10,11,12,13], while the dielectric constant of the saturate fraction ranges from 1.58 to 1.91 [11,14,15]. Despite its varying dielectric properties, heavy oil demonstrates some microwave absorption capabilities. To enhance this, some propose introducing a superior microwave-absorbing material that is cheap and compatible with oil such as carbon [16]. Given its dielectric constant of roughly 8.0 [17], carbon’s absorption capacity is nearly quadruple that of crude oil. This addition could potentially facilitate thermal cracking at temperatures as low as 150 °C [12].

Microwave-induced selective heating leads to an uneven temperature distribution or the formation of “hotspots” [18]. These hotspots, characterized by temperature gradients within the material, expedite the breakdown of complex hydrocarbons into smaller molecules, thereby reducing viscosity. Localized areas within heavy oil can exhibit temperatures 100–200 °C higher than the bulk fluid under microwave radiation, causing localized overheating [19]. The formation of these hotspots could potentially accelerate cracking reactions compared to traditional heating methods.

This paper aims to bridge the gap in the existing literature by exploring the effects of different carbon susceptors on microwave utilization in bitumen upgrading. The study investigates the influence of various parameters, like microwaving time, operating temperature, carbon additive concentration, and agitation rate, on the extent of reaction and viscosity reduction. The effects of different carbon susceptors, such as activated carbon, graphite, biochar, and coke, on the viscosity of Canadian bitumen oil under microwave irradiation are also assessed. The goal is to optimize physical operating conditions to better understand their impact on bitumen’s physical and chemical properties and hydrocarbon fraction composition.

## 2. Results

### 2.1. Effects of Carbon Susceptors on Bitumen Upgrading

#### 2.1.1. The Role of Carbon Susceptors in Microwave Absorption and Heating Rates

**Figure 2:**
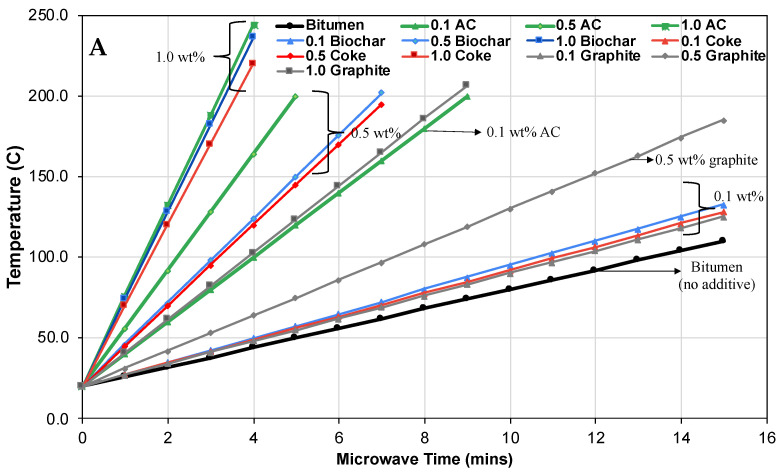

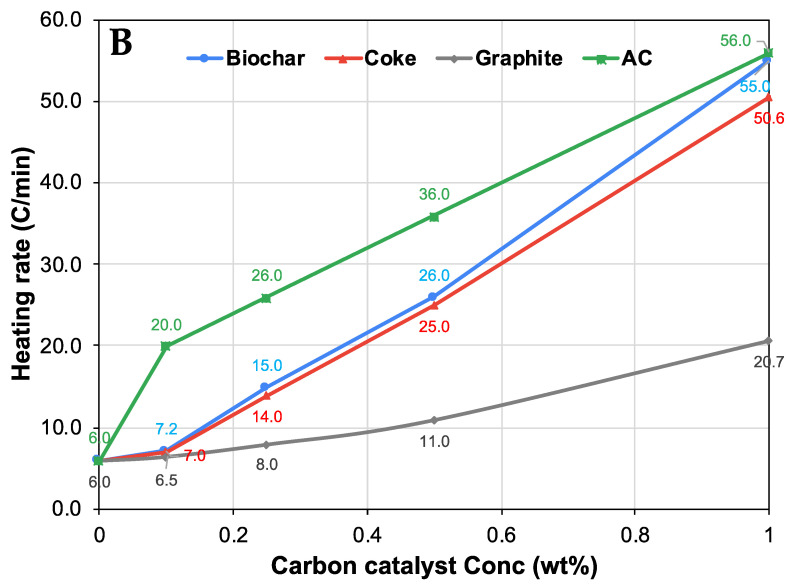
(**A**) Bitumen recorded temperatures with carbon susceptors at different concentrations. (**B**) The effect of different carbon susceptor concentrations on heating rates.

The impact of the carbon susceptors on the microwave heating rate and the time taken to reach a target temperature of 200 °C in oil samples is illustrated in Figure 2A,B. The heating rate under microwave irradiation depends on the oil sample’s ability to absorb and convert microwave energy into heat. Four types of carbon susceptors—activated carbon, coke, graphite, and biochar—at concentrations ranging from 0 to 1.0 wt%, were tested in bitumen samples to evaluate their effects on heating rates. The results, demonstrating different absorption capabilities among the susceptors, can be attributed to factors such as the dipole moment of a molecule, the contact surface area, and the elemental composition [16].

In the absence of a carbon susceptor, bitumen reached a peak temperature of 125 °C after 15 min of microwave heating (heating rate of 6 °C/min). Conversely, the addition of a small quantity of 0.1 wt% of activated carbon (AC) allowed the bitumen sample to hit the 200 °C mark within 9 min, enhancing the heating rate to 20.8 °C/min. By increasing the AC concentration, the time to reach 200 °C was significantly reduced, taking only 5 min at 0.5 wt% and 4 min at 1.0 wt% AC.

Biochar and coke exhibited similar trends, needing a minimum of 4 min to reach 200 °C at 1 wt%. At a lower concentration of 0.1 wt%, however, it took about 25 min to reach the desired temperature. At 0.5 wt%, both biochar and coke reached 200 °C in around 7–8 min, corresponding to a heating rate of 25 °C/min, comparable to 0.1 wt% AC. Graphite, in comparison, showed the least absorption capabilities. Its most efficient heating rate of 21 °C/min was achieved at 1 wt%, taking approximately 8.8 min to reach 200 °C. This rate was comparable to 0.1 wt% AC or 0.5 wt% of either biochar or coke. At 0.5 wt% and 0.1 wt%, graphite needed roughly 16 and 25 min of microwave heating, respectively.

#### 2.1.2. The Effects of Carbon Susceptors on Bitumen’s Viscosity

**Figure 3:**
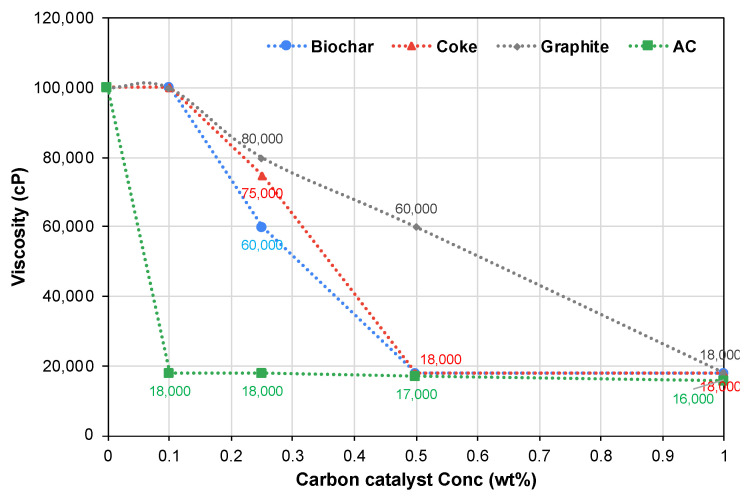
The effects of carbon susceptor concentrations on bitumen’s viscosity after microwaving at 150 °C for 10 min.

Figure 3 outlines the viscosity changes for the upgraded oil samples at different carbon susceptor concentrations under microwave irradiation. After reaching 150 °C, or the maximum feasible temperature for samples with lower carbon susceptor concentrations, the samples were maintained at that temperature for 10 min. Viscosity measurements were taken three times for each condition at room temperature (20 °C), and the average values are presented in Figure 3.

Remarkably, with the assistance of a carbon susceptor under microwave irradiation for merely 10 min, bitumen’s viscosity dropped from its initial value of 100,000 cP to 18,000 cP. However, this significant reduction was achieved at varying carbon susceptor concentrations. Only 0.1 wt% of activated carbon (AC) was needed for the maximum viscosity reduction, with any further AC concentration increase failing to significantly alter the final viscosity, making 0.1 wt% the optimum concentration. Contrastingly, the maximum viscosity reduction in the other bitumen samples necessitated a 0.5 wt% concentration of either biochar or coke. As with AC, any concentration increase beyond the optimum had no impact on the final viscosity, rendering 0.5 wt% the optimal concentration for both biochar and coke.

Graphite, however, required a higher concentration—1.0 wt%—for maximum viscosity reduction, double that of biochar or coke and ten times that of AC. The variation in the optimum susceptor concentrations could be attributed to differing trace metal contents within the carbon particles, impacting their catalytic and microwave absorption abilities, or to the surface area and pore volume associated with each particle, affecting the susceptors’ surface properties and microwave radiation absorption abilities. These aspects are further explored in subsequent sections of this paper.

#### 2.1.3. The Effects of Carbon Susceptors on Bitumen’s SARA Fractions

The SARA (Saturates, Aromatics, Resins, and Asphaltenes) compositions of both the original bitumen sample and the upgraded samples, after 10 min of microwave heating at 150 °C, are presented in Table 1. Only the optimum concentrations of each of the four susceptors (0.1 wt% AC, 0.5 wt% biochar, 0.5 wt% coke, and 1.0 wt% graphite), required to achieve the maximum viscosity reduction, were considered. Notably, these optimum concentrations offered almost equivalent heating rates of 21 °C/min for the bitumen samples.

A consistent observation across the upgraded samples was a substantial increase in the saturate fraction, which increased from the original 18.5% to around 30% within a brief span of 10 min of microwave exposure. This remarkable transformation underscores the susceptibility of resin and asphaltene constituents, which, being rich in S, N, and O elements, are the most common polar compounds, to microwave radiation, crucial in facilitating electromagnetic field heating [21]. Interestingly, the asphaltene content experienced a marginal decrease, attributed to a limited extent of cracking reactions probably due to the limited reaction time. Conversely, the resin and aromatic fractions displayed significant reductions, indicative of substantial cracking into smaller, saturated molecules during the microwave heating process. For instance, in the AC sample, the aromatic and resin contents were reduced by about 7.5 wt% and 9.7 wt%, respectively. These findings align with those of studies such as Zhang et al.’s [12], which reported that, out of the SARA fractions, resins and aromatics had the most significant impact on the dielectric loss factor in heavy oils, while saturates and asphaltenes had a minor impact. Contrary to previous beliefs, asphaltenes, despite being a primary polar component, were found to have a minor contribution to the dielectric loss. This behavior can be attributed to the low molecular mobility of the molecules, inhibiting their response to the electromagnetic field at microwave frequencies.

In terms of carbon susceptors, the 0.1 wt% activated carbon (AC) sample demonstrated the greatest increase in saturates (from 18.5 wt% to 31.2 wt%) alongside the most significant reduction in viscosity (from 300,000 cP to 18,000 cP) when compared with the other three susceptors. At the same time, there was a slight reduction in aromatics, with resins and asphaltenes witnessing larger decreases. This highlights AC’s efficiency, owing to its superior surface area and porosity, in absorbing microwaves and promoting the thermal cracking of heavier fractions into lighter ones. From a density perspective, all upgraded samples recorded a drop from the original 1020 kg/m^3^ to 1000 kg/m^3^, indicative of the successful thermal cracking of heavier fractions, consequently reducing the overall weight of the bitumen. When compared with conventional upgrading methods, microwave upgrading with carbon susceptors appears to exhibit superior control over the hydrocarbon composition of the bitumen. Traditional methods, such as coking or hydroprocessing, typically necessitate higher temperatures and pressures and are associated with substantial energy consumption and greenhouse gas emissions. Furthermore, these methods may yield a higher proportion of undesired products, like coke or gas. Conversely, microwave upgrading seems to augment the desirable saturate fraction and substantially curb bitumen’s viscosity, resulting in a more efficient and environmentally benign upgrading process.

#### 2.1.4. The Role of Carbon Susceptors in Desulfurization of Bitumen

Table 2 outlines the sulfur content of the bitumen samples, which were subjected to microwave heating at 150 °C for 10 min with the aid of four carbon susceptors at their optimum concentrations. The results indicate a substantial reduction in sulfur content, by a factor of 21–24%, across all upgraded samples. Notably, the sample containing 0.1 wt% activated carbon (AC) facilitated the greatest sulfur reduction compared to the other carbon susceptors.

This significant reduction can be attributed to the occurrence of cracking reactions in or near the hotspots created by the carbon susceptors during microwave heating. The presence of these hotspots increases the likelihood of heavier components, typically rich in sulfur, undergoing cracking. This process can break C-S bonds, allowing for the release of sulfur in the form of H_2_S, which results in a lower sulfur content.

The varying desulfurization rates among the different carbon susceptors are likely related to their individual properties. Specifically, AC, even at a lower concentration, proved the most efficient, possibly due to its high surface area and porosity, enhancing microwave absorption and interaction with sulfur compounds. Conversely, despite a higher concentration, graphite achieved slightly lower desulfurization, potentially due to its lower porosity and surface area. Biochar and coke exhibited intermediate performance, likely due to their properties falling between those of AC and graphite. These findings underscore the role of carbon susceptors in enhancing microwave-induced desulfurization in bitumen. Nonetheless, a more in-depth investigation is necessary to understand the influence of carbon susceptors’ surface properties on microwave absorption, a topic that is further investigated in the subsequent section.

### 2.2. Surface Properties of Carbon Susceptors and their Effects on Microwave Absorption

#### 2.2.1. Effect of Trace Metal Content

The disparity in heating rates among the different carbon susceptors was initially explored through a leaching process aimed at demineralizing carbon particles of any trace metal components. The procedure adopted was similar to that outlined in Reference [22]. This involved submerging 20 g of activated carbon in 500 mL of 0.1 mol/L HCl, ensuring even dissolution through gentle stirring, and allowing it to rest for 2 h at 20 °C. The resulting solution was then filtered, with the carbon residue thoroughly rinsed with distilled water until neutralization. The leached carbon powder, nearly pure at 99.99% carbon and less than 0.01% trace elements, was dried in an oven at 120 °C to eliminate any residual moisture.

When the leached AC was incorporated into bitumen for microwave heating, both the heating rate and viscosity values were measured. The findings revealed no discernible difference between the leached and un-leached AC samples in terms of heating rates or viscosity reduction across the different concentrations. A detailed account of this study can be found in Section S-3 of the attached Supplementary Material. Based on these observations, it was concluded that the trace metal content in the carbon susceptors did not influence either the catalytic effect or microwave absorption abilities. Therefore, the variation in heating rates might primarily be attributed to a different surface property. This important discovery directs the path for future investigations.

#### 2.2.2. Impact of Surface Area and Porosity

Investigations from several studies on porous carbon materials suggest that such structures, by providing alternative pathways for incident waves, can enhance electromagnetic wave absorption [23]. The porous nature of these materials enables multiple instances of absorption and the scattering of electromagnetic waves, subsequently converting them into heat. This process enhances the dielectric loss factor of the absorbing material, a parameter signifying the material’s efficiency in converting radiation into heat. An increased pore volume decreases effective permittivity and enhances connectivity with free space. Such properties allow electromagnetic waves to penetrate porous absorbers more efficiently and remain trapped for extended periods. Given these considerations, this study targeted the surface area and porosity of the carbon susceptors. All four were analyzed using the BET method, with the summarized results presented in Table 3.

The activated carbon demonstrated a significantly higher surface area and pore volume than the other carbon susceptors, explaining why a mere 0.1% AC was needed to achieve the maximum thermal conversion and viscosity reduction of bitumen. Conversely, higher amounts were necessary with biochar, coke, and graphite.

To further substantiate the proposed relationship of surface area and porosity with microwave absorption, the initially used biochar was further activated using KOH, enhancing its surface area and porosity beyond those of the activated carbon. This activated biochar was then blended with bitumen at various concentrations for microwave absorption testing. The performance of the activated biochar surpassed that of activated carbon, achieving a heating rate of 20.8 °C/min and a maximum viscosity reduction at just 0.05 wt%. This result suggests the new optimal concentration of activated biochar is 0.05 wt%, half that of AC. Conclusively, this investigation affirms a direct correlation between the surface area and porosity of the carbon susceptors and their microwave absorption and heating rate.

Further exploration was conducted to corroborate the established correlation between the surface area of carbon particles and their microwave absorption capabilities. Each type of carbon particle was placed individually in a quartz tube and directly subjected to microwave radiation without a solvent. This experiment aimed to expose potential differences in microwave absorption properties among the carbon susceptors and to compare hotspot temperatures with the previously measured bulk temperatures of the bitumen mixture. The outcomes of this investigation are consolidated in Table 4.

The achieved maximum temperatures underscore the assertion that carbon particles with larger surface areas and greater porosities exhibit enhanced microwave absorbance. One theory postulates that chars or cokes, due to their abundant delocalized electrons forming graphic structures, possess superior microwave absorption qualities [24]. Furthermore, the presence of micropores within the carbon particles can make the susceptor an effective medium for microwave absorption, enhancing impedance matching and controlling effective permittivity. This theory finds support from Negi et al. [25], who demonstrated that the porous structure of the susceptor particle facilitates high impedance matching and the subsequent attenuation of microwave radiation.

This investigation also highlights the significant discrepancy between local and bulk temperatures. With just 60 s of microwave heating, the temperature of activated carbon and activated biochar can reach 750 °C and 800 °C, respectively—high enough to fracture nearly all hydrocarbon bonds within the bitumen mixture. However, within the larger fluid body, heat is quickly dissipated from these localized hotspots, resulting in considerably lower overall bulk temperatures. Therefore, only bitumen fractions close to these hotspots undergo high-temperature exposure and consequent cracking.

### 2.3. Evaluation of Microwave Irradiation Operational Parameters on Upgrading Bitumen

#### 2.3.1. Effect of Microwave Irradiation Time

In this investigation, the effect of varying irradiation times on the characteristics and quality of the upgraded oil was evaluated. Activated carbon (AC), given its superior surface area and porosity and, hence, better microwave absorbing abilities, as established in prior sections, was selected as the best carbon-based susceptor for the subsequent investigation. A concentration of 0.1 wt% AC was identified as the optimum concentration, capable of inducing the maximum viscosity reduction. This concentration was maintained, while other operational parameters underwent alteration. Firstly, the impact of microwaving duration was tested over a range of 0–20 min while maintaining a reaction temperature of 150 °C, which had been previously identified as the minimum required to induce significant changes in bitumen properties.

**Figure 4:**
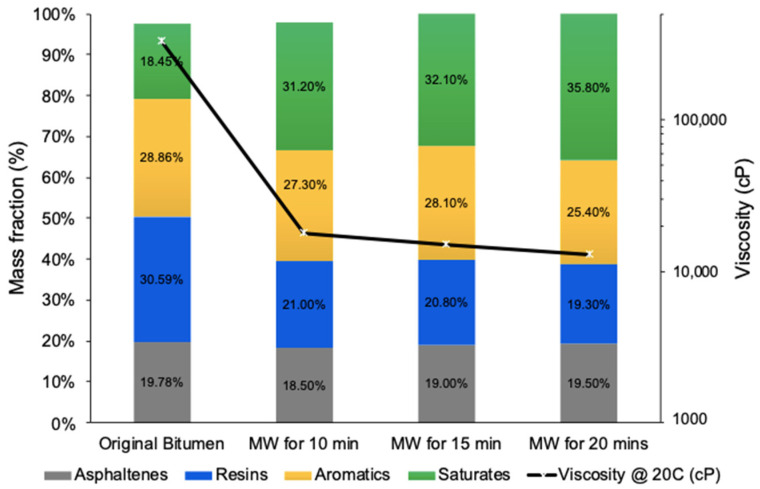
The effect of microwave irradiation time at the fixed temperature of 150 °C on the viscosity reduction and SARA components of upgraded oil with 0.1 wt% AC.

Figure 4 depicts the correlation between viscosity and SARA compositional changes concerning microwaving time. An irradiation duration of 10 min resulted in a dramatic viscosity reduction from 100,000 cP to 18,000 cP, translating to an 82% decrease. Extending the microwaving time by an additional 5 min (totaling 15 min) incrementally enhanced viscosity reduction, reaching a new viscosity of 15,000 cP and an overall reduction of 85%. Further extending the microwaving time to 20 min resulted in a viscosity of 13,000 cP and a maximum reduction of 87%. These data suggest that the majority of thermal cracking reactions transpire within the initial 10 min of microwave irradiation, with any extension beyond this duration offering negligible improvements as viscosity values plateau.

Similarly, an analysis of SARA fractions within the same microwaving times revealed a pattern. Within the initial 10 min reaction window, the maximum reduction in resins and aromatics and the maximum increase in saturates by 72% were observed. A further extension of the microwave irradiation time beyond this resulted in marginal improvements in hydrocarbon fractions until they plateaued at 20 min. The asphaltene fraction exhibited a minor decrease in the first 10 min, and then it remained constant, showing no further changes.

#### 2.3.2. Effect of Maximum Microwaving Temperature

**Figure 5:**
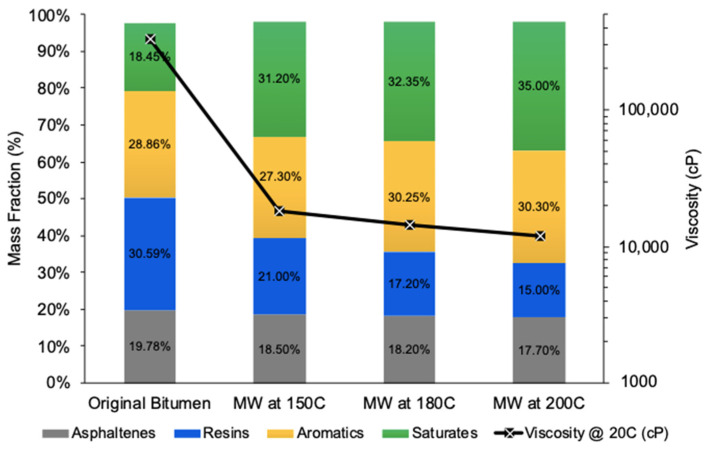
The effect of microwave (MW) reaction temperature at a fixed time of 10 min on the viscosity reduction and SARA components of upgraded oil with 0.1 wt% AC.

In this phase of the investigation, the impact of different maximum microwave temperatures on the upgraded oil was evaluated. The maximum reaction temperature was varied within the range of 150–200 °C, while the reaction time was fixed at 10 min. Figure 5 illustrates the viscosity and SARA compositional changes in relation to the microwave temperature. The findings indicate that a reaction temperature of 150 °C facilitated a reduction in bitumen viscosity from 300,000 cP to 18,000 cP, signifying a 94% decrease. Increasing the maximum reaction temperature to 180 °C further reduced viscosity to 14,500 cP, marking a 95.2% reduction. Finally, pushing the microwaving temperature to 200 °C led to a viscosity of 11,000 cP, equating to a final viscosity reduction of approximately 96%.

**Figure 6:**
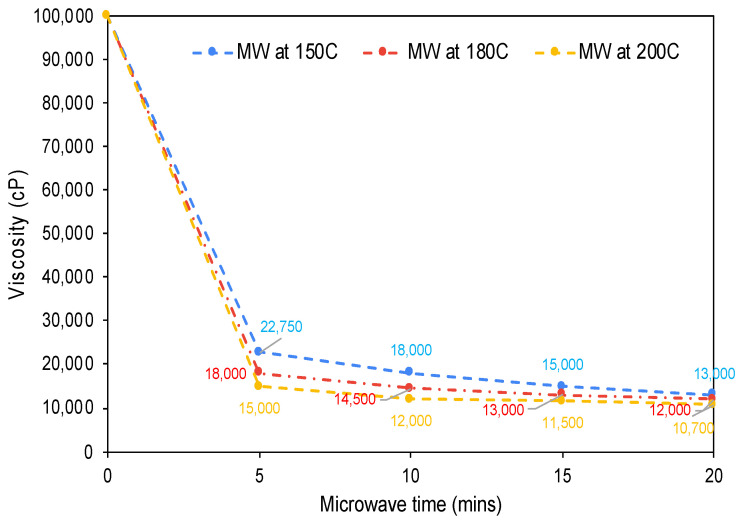
Summary of the combined effect of microwave reaction time and temperature on the viscosity reduction of upgraded oil with 0.1 wt% AC.

Subsequently, the combined effect of varying both the maximum microwaving temperature and the heating time was examined. The parameters adjusted were the temperature, within the range of 150–200 °C, and the reaction time, from 0 to 20 min. Figure 6 encapsulates the viscosity changes as a function of both the microwave temperature and time. It shows that the maximum viscosity reduction occurred in the first 5 min of microwave heating at all tested temperatures. This was then followed by a deceleration in the reduction rate over the next 15 min. The optimal viscosity reduction was achieved under any one of the following conditions: microwaving at 200 °C for 10 min, at 180 °C for 15 min, or at 150 °C for 20 min. Any increase beyond these parameters did not significantly alter the viscosity of bitumen or the compositional structure of the oil samples’ hydrocarbon fractions.

Furthermore, the sulfur content was assessed in the bitumen samples microwaved at varying temperatures and times, using XRF, with an error bar of (+/−0.01%). Table 5 summarizes the average sulfur results. Most tested microwaving conditions exhibited a similar effect on bitumen desulfurization, with a minor improvement as either temperature or time increased. The maximum sulfur reduction was reported after microwaving bitumen at the highest irradiation of 200 °C for 20 min, achieving a 5% sulfur reduction and reaching a minimum content of 4.45 wt%.

#### 2.3.3. Impact of the Stirring Rate

**Figure 7:**
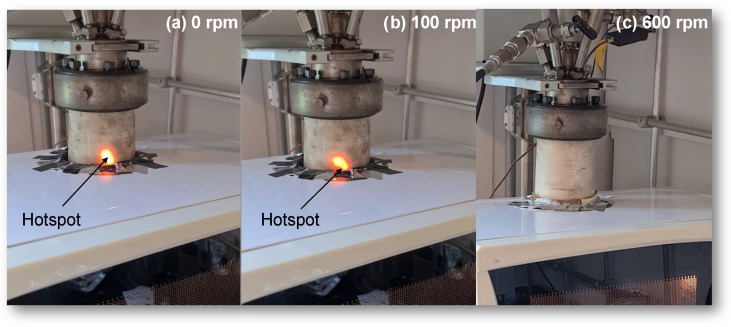
Real-time illustration of the hotspots at various stirring rates: (**a**) 0 rpm, (**b**) 100 rpm, and (**c**) 600 rpm.

In this section, the effect of the stirring rate on both the heating progression and the extent of cracking reactions is explored. The visibility of hotspots within the light-colored PTFE reactor provided real-time observations during microwave irradiation at different stirring rates, as depicted in Figure 7. Without stirring (0 rpm) or with slow stirring (100 rpm), the formation of orange-colored hotspots was conspicuous. However, a surge in the stirring rate gradually diminished both the number and intensity of these hotspots, which were rendered undetectable at a rapid stirring rate of 600 rpm. The emergence of such hotspots is due to microwave radiation reflection off the surface of the activated carbon within the oil, a phenomenon also observed in Horikoshi et al.’s study [26]. The temperature of these hotspots can reach an impressive 900 °C, as noted in certain studies [26].

**Figure 8:**
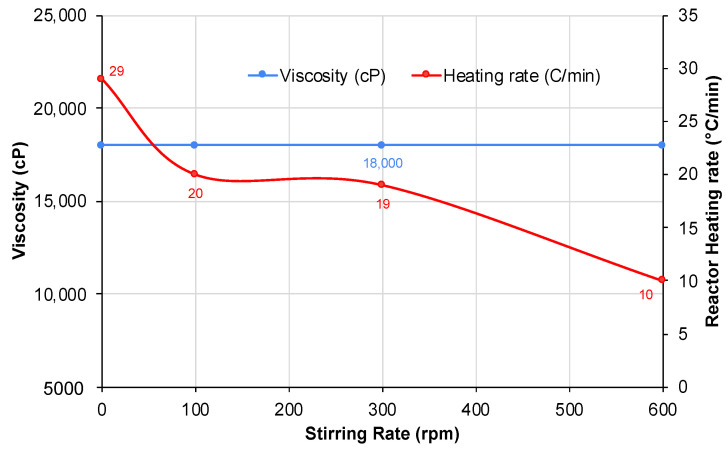
The effect of the stirring rate on the viscosity and heating rates.

The stirring rate’s impact on the heating progression of the bitumen samples is illustrated in Figure 8. An increased stirring rate resulted in a slower heating progression. While the heating rate remained relatively constant within the slow-to-medium stirring speeds (100–300 rpm), it experienced a significant drop when the stirring rate exceeded 300 rpm, bottoming at a heating rate of 10 °C/min at 600 rpm. The rapid rotation probably limits the opportunity for microwave radiation to engage with the carbon surface, reducing absorption and reflection, and leading to a longer heating period.

However, stirring also facilitates a more uniform temperature profile within the bulk fluid and increases the likelihood of carbon particles interacting with bitumen’s heavier fractions. Therefore, a slow stirring rate within 100–300 rpm is recommended, as employed in this study’s experimental runs. Importantly, irrespective of the heating or stirring rates, the final viscosity remained consistent, suggesting that the carbon susceptor’s absorption capacity was adequate for inducing cracking reactions. As a consequence, the stirring rate appears to affect the rate of reaction rather than the extent of the reaction, providing that the sample receives sufficient heating.

## 3. Materials and Methods

### 3.1. Materials Used

The main feedstock for this study was oil sand bitumen sourced from the Athabasca reservoirs in Alberta. The key physical properties of this bitumen are provided in Table 6. Viscosities were measured using the ROTAVISC rotational viscometer from IKA, with a measurement range of 100–4,000,000 cP and a repeatability of 1%, following the ASTM D4402 standards [27]. Density measurements were conducted using a specific gravity hydrometer from Fisher Scientific. A Saturate, Aromatic, Resin, and Asphaltene (SARA) analysis was performed to classify the crude oil constituents based on their polarizability and polarity, following the ASTM D2007 standard process [20].

All carbon-based susceptors used in this study, except for biochar, were procured from Fisher Scientific, each with a purity > 99% and a negligible ash and trace metal content. This study employed four distinct carbon-based materials. Firstly, the commercially available DARCO G-60 activated carbon, a highly porous powder with a mesh size between 100 and 325 (149–45 µm), was used. Secondly, synthetic graphite powder was utilized, having a composition of 99% carbon and 0.2% ash and a mesh size ranging from 100 to 200 (149 to 74 µm). The third material was calcined petroleum coke powder, a crystalline structured substance with a mesh size of approximately 325 (~45 µm). Lastly, this study incorporated synthesized biochar, a product of pyrolyzing pinewood sawdust, a waste biomass that is readily available, at 300 °C for 30 min with K_2_CO_3_ as a catalyst. The resulting biochar exhibited a mesh size between 100 and 325 (149 and 45 µm).

### 3.2. Experimental Design

Microwave irradiation experiments were conducted using a standard commercial microwave oven, specifically the Danby 0.7 cu. ft. Countertop Microwave. This microwave oven operates at a power of 700 watts. The choice of a commercially available microwave oven was driven by the aim of this study to explore the feasibility of microwave-assisted bitumen upgrading using readily accessible and cost-effective equipment. This choice presents a realistic scenario for potential applications in smaller-scale or preliminary industrial trials.

**Figure 9:**
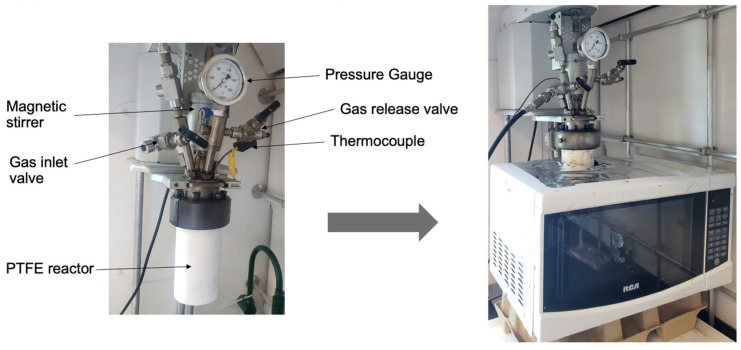
Pictures of the experimental setup used for microwave upgrading.

Moreover, it is important to note that, in microwave-assisted processes, the reactor’s fabrication material plays a critical role due to its interaction with microwaves. Therefore, Polytetrafluoroethylene (PTFE), a microwave-transparent plastic, was chosen for this study over other potential materials, such as glass or ceramic. PTFE offers superior chemical resistance, physical durability, and ease of cleaning, in addition to its resilience to sudden thermal shocks. While PTFE starts to degrade at temperatures higher than 260 °C, this is not a concern for this study, as we made sure that the oil temperature did not exceed 200 °C, providing a safety margin of 60 °C. Temperature measurements during the microwave irradiation process were conducted using a carefully shielded fiber optic temperature sensor (FOTS). The sensor was precisely positioned within the PTFE reactor to ensure contact with the bitumen mixture, without touching the reactor’s sides. Before each experiment, the sensor was calibrated, with the temperature readings displayed in real-time during the irradiation process. This ensured that the microwaves interacted exclusively with the bitumen mixture, not the sensor. The agitation method involved a metallic stirrer, typically not recommended due to the potential for metal–microwave interactions. However, some measures were taken to mitigate such interactions. The stirrer was aligned parallel to the electromagnetic field, minimizing potential interference. In addition, the stirrer was designed with a smaller diameter and smoother surface to decrease the risk of arcing and heating by reducing the exposed surface area to the microwave field. Pictures of the experimental setup used in the microwave partial upgrading series of tests are shown in Figure 9.

### 3.3. Sample Processing Method

Approximately 50 g of bitumen was initially heated in a hot water bath until flowable (approximately 80 °C), and then it was introduced into the PTFE reactor. This was combined with carbon additives and agitated at 300 rpm for 10 min to ensure a homogeneous mixture. The bitumen sample was subsequently heated using the commercial microwave, operating at 2.5 GHz and 700 W. To maintain a non-reactive environment, the reactor assembly was filled with nitrogen gas. Post-heating, the upgraded bitumen was extracted from the PTFE reactor, and its physical properties—viscosity, density, and SARA hydrocarbon composition—were measured. All microwave upgrading reactions yielded approximately 97–98% liquid, with no observed coke formation.

The initial biochar utilized in this study was derived from the pyrolysis of pinewood biomass. However, the inherent surface area and porosity resulting from the pyrolysis process were relatively low; therefore, an activation process was used to elevate these parameters. This was crucial to equate the characteristics of the biochar to those of the commercially available activated carbon, thereby enabling a comparative study on the influence of the surface area and porosity on the heating efficiency of microwave irradiation. The activation procedure was composed of several steps. Initially, 6 g of biochar was amalgamated with 6 g of KOH and 20 mL of pure water within a centrifuge tube. This mixture was then agitated for approximately 2 h. Following this, the solution underwent filtration, with the biochar residue subjected to washing using a diluted acid solution until neutrality was attained. After neutralization, the residue was dehydrated at 80 °C for 48 h. To complete the activation, the dried biochar was placed within a ceramic crucible and exposed to a heating rate of 10 °C/min until a temperature of 800 °C was reached. This temperature was maintained for 2 h under an inert atmosphere within an activation unit.

### 3.4. Characterization Methods

The Nova 1200E BET analyzer from QuantaChrome was used for a surface area analysis of the four carbon-based susceptors. Before measurements, the samples were degassed at 150 °C for over 3 h in a vacuum. The BET nitrogen adsorption and desorption method was used at 77°K to determine the specific surface area and pore size distribution, following the BET and BJH equations, respectively.

The sulfur percentage in hydrocarbons was determined following the ASTM D4294 standard [28]. The sample was injected into a measurement cell and exposed to an X-ray tube beam. The emitted characteristic X radiation was measured, and the collected count was compared with that from calibration standards to determine the sulfur concentration in mass percent. Three concentration ranges were covered by the calibration samples: 0.0 to 0.1 mass%, 0.1 to 1.0 mass%, and 1.0 to 5.0 mass% sulfur.

## 4. Discussion of the Key Results

This study provides critical insights into microwave-assisted bitumen upgrading leveraging carbon-based particles as microwave susceptors. The key observations encompass the relationship between the characteristics of the carbon susceptors and microwave absorbance, the effect of the microwave irradiation parameters on upgraded oil, and the stirring rate’s influence on the process.

Activated carbon proved to be the most efficient susceptor owing to its superior surface area and porosity. Furthermore, microwave duration and temperature emerged as critical factors in decreasing bitumen’s viscosity, with the majority of thermal cracking reactions occurring within the initial 10 min of microwave exposure. Additionally, the stirring rate was found to significantly influence the heating rate—a rise in the stirring rate resulted in a decrease in the heating rate. However, this did not affect the extent of the reaction, implying that only the reaction rate was influenced.

Despite the advantages of incorporating carbon particles in the bitumen microwave partial upgrading process, this does introduce some potential operational challenges. Mainly among these is the possible build-up of carbon particles in the refineries’ processing equipment, which could hinder operational efficiency or even cause equipment damage. Therefore, several preventative measures can be implemented, such as post-processing separation methods to extract carbon particles from the upgraded oil. Techniques like centrifugation, filtration, or sedimentation can be employed based on the carbon materials’ particle size and characteristics. Given that carbon susceptors’ particle size usually falls within the micron or millimeter range, their separation should be manageable. An innovative approach involving the use of magnetic carbon susceptors could further simplify the separation process. Applying a magnetic field could allow for the easy separation of these susceptors from the oil, mitigating the risks linked to particle accumulation.

The transformation of bitumen properties through microwave upgrading hinges on several mechanisms, mainly the thermal and chemical influences of microwave irradiation and the role of the carbon susceptors employed in the process. Microwave irradiation is known to trigger certain chemical reactions, including the catalytic cleavage of C-S and C-C bonds, which were witnessed, as the sulfur content and the viscosity of bitumen were reduced on average by 24% and 94%, respectively. Furthermore, carbon susceptors, characterized by their larger surface area and porosity, can absorb more microwaves, generating localized hotspots within bitumen, resulting in more efficient hydrocarbon cracking. The greater surface area also presents more sites for catalysis.

Furthermore, to visualize the significance of employing microwave radiation as a partial upgrading technique, the Walther equations, as utilized by Arno de Klerk in [29], were used to accurately calculate the diluent volume required for the partially upgraded bitumen post-microwave treatment. The equations are mentioned below and were employed to meet target pipeline specifications of 350 cSt and 940 kg/m^3^:

(1) $$\log\left(\log\left(v_{m}+0.7\right)\right)={\;w}_{1}*\;\log\left(\log\left(v_{1}+0.7\right)\right)+{\;w}_{2}*\;\log\left(\log\left(v_{2}+0.7\right)\right)$$

(2) $$\rho_{m}={\left[\frac{w_{1}}{\rho_{1}}+\frac{w_{2}}{\rho_{2}}\right]}^{-1}$$

Here, v_1_ is the viscosity of the upgraded bitumen; v_2_ is the viscosity of the diluent; w_1_ and w_2_ are the weight fractions of bitumen and the diluent, respectively; v_m_ is the viscosity of the mixture (bitumen + diluent); and $\rho_{m}$ is the density of the mixture. It should be noted that the density of the diluent was assumed to be 642 kg/m^3^, and its dynamic viscosity was taken to be 0.5 cP at 25 °C, equivalent to 0.8 cSt (kinematic viscosity).

Assuming light naphtha as the main diluent with the density and viscosity values mentioned above, the calculations revealed a significant reduction in the required diluent volume, from the initial 30% to just 20% post-microwave treatment. This requirement can reduce the oil’s viscosity and density from 10,000 cSt and 1000 kg/m^3^ post-microwave irradiation to approximately 350 cSt and 940 kg/m^3^ as per the pipeline specifications. This significant saving, marking a 10% decrease, was achieved merely by subjecting bitumen to microwave radiation for 20 min at 200 °C. These findings highlight the efficiency of microwave radiation as a means to enhance bitumen’s viscosity and density properties, ultimately leading to a more efficient and cost-effective use of diluents in the bitumen upgrading process.

In addition to the technical advancements demonstrated in this study, a preliminary analysis was conducted, and it suggested that the microwave-assisted bitumen upgrading process could offer significant cost and environmental benefits compared to traditional thermal cracking methods like visbreaking. The microwave irradiation technique, particularly with activated carbon as a susceptor, shows potential for higher energy efficiency and reduced operational costs due to lower energy consumption and shorter processing times, given that the initial capital costs of both processes are assumed to be the same. Additionally, the quality and yield of the upgraded bitumen could potentially enhance its market value, especially considering the significant viscosity reduction and improved SARA fractions. From an environmental standpoint, microwave irradiation is considered to be a greener alternative, primarily due to its lower greenhouse gas emissions. This is attributed to the process’s energy efficiency and the potential for cleaner energy sources. Furthermore, the method might produce fewer or less harmful byproducts, contributing to a reduced environmental footprint. While these observations are based on theoretical considerations and necessitate further empirical validation, they underscore the potential of microwave irradiation as a more sustainable and cost-effective approach to bitumen partial upgrading. Therefore, detailed empirical studies or pilot-scale operations would still be necessary to provide concrete data and validate these assumptions.

Finally, while this study provided significant insights into microwave-assisted bitumen upgrading, it also opens several avenues for future research. Paramount among these is the exploration of the scalability and industrial applicability of this method. Further studies are essential to assess the operational efficiency and economic viability of scaling up the microwave irradiation process for industrial-scale applications. Additionally, there is a need for long-term performance and reliability studies to evaluate the durability of the equipment and the consistency of upgraded bitumen quality over extended periods. Environmental impact assessments are also crucial, particularly focusing on greenhouse gas emissions and energy consumption, to comprehensively validate the environmental benefits of this technology. Technological advancements in microwave irradiation equipment and techniques should also be a focus, aiming at improving efficiency, reducing operational costs, and enhancing overall safety.

## 5. Conclusions

The investigation carried out in this study thoroughly examined the effect of microwave irradiation, supplemented by various carbon susceptors, on the partial upgrading of bitumen. The findings indicate that unassisted microwave irradiation of bitumen delivers a slow heating rate with a negligible impact on viscosity reduction. However, when supplemented with 0.1 wt% activated carbon, a brief 10 min microwave treatment effectively raised the oil’s bulk temperature to 200 °C at a rate of 20.8 °C/min, resulting in over a 90% decrease in bitumen viscosity. Extending the microwave exposure to 20 min at 150 °C with 0.1 wt% activated carbon induced additional cracking reactions, resulting in a further 40% reduction in viscosity to 18,000 cp. Furthermore, increasing the maximum microwave temperature from 150 °C to 200 °C with activated carbon stimulated additional cracking within the resin and aromatic fractions, achieving a maximum viscosity reduction. In addition to that, it was shown that the addition of just 0.1 wt% activated carbon within the bitumen blend was able to absorb the microwave radiation, creating hotspots that trigger the thermal cracking of highly polar resins and aromatic fractions into less polar, lighter saturates, with a liquid yield of over 97%. Superior microwave absorption capabilities of carbon susceptors with larger surface areas and higher porosity were also observed. Surprisingly, increasing the stirring rate during microwave irradiation resulted in a slower heating rate and faster cooling of the reaction mixture, with no significant impact on viscosity changes or the extent of the reaction. Thus, this study’s results suggest that activated carbon particles can serve as an effective additive for microwave partial upgrading. This approach can achieve a reduction in bitumen viscosity of up to 96%, at considerably lower temperatures and reaction times, with less GHG emissions, and overall lower operational costs than conventional upgrading methods.

## Figures and Tables

**Table 1:** SARA composition of oil samples before and after 10 min microwave heating at 150 °C.

	Hydrocarbon Composition (wt%)					
Oil Samples	Saturates	Aromatics	Resins	Asphaltenes	Density (kg/m^3^)	Viscosity (cP)
Original Bitumen	18.5	28.9	30.6	19.8	1020	300,000
Bitumen with 0.1 wt% AC	31.2	27.3	21.0	18.5	1000	18,000
Bitumen with 0.5 wt% Biochar	30.6	25.7	22.6	19.1	1000	18,400
Bitumen with 0.5 wt% Coke	30.8	25.1	23.0	19.1	1000	18,600
Bitumen with 1 wt% Graphite	28.8	26.4	23.4	19.4	1000	19,000

Note: When bitumen samples are analyzed using clay column chromatography to determine their group composition, the sum of the percentages of the light and heavy components does not equal 100% since some nonhydrocarbon reserves remain in the column. The total mass of all the recovered fractions is equal to 97.68% (which still satisfies the ASTM-2007 [20] requirement of being >97%).

**Table 2:** The sulfur content of microwaved bitumen measured using XRF.

Sample	Sulfur Content (wt%)	Desulfurization (%)
Original Bitumen	4.67	-
Upgraded with 0.1 wt% AC	3.54	24.2
Upgraded with 0.5 wt% Biochar	3.63	22.3
Upgraded with 0.5 wt% Coke	3.66	21.6
Upgraded with 1.0 wt% Graphite	3.58	23.3

**Table 3:** BET results of the four carbon susceptors.

Susceptor	Multi-Point BET Specific Surface Area (m^2^/g)	Total Pore Volume (cm^3^/g)	Average Pore Size (nm)
Activated Carbon	415.46	1.290	6.22
Biochar	12.01	0.023	6.50
Coke	11.39	0.038	6.72
Graphite	4.16	0.008	3.98
Activated Biochar	561.3	2.860	6.55

**Table 4:** The maximum temperature reached by carbon susceptors via direct microwave heating.

Particle Temperature (°C)					
Microwaving Time (s)	Activated Carbon	Biochar	Coke	Graphite	Activated Biochar
10	125	100	108	82	135
20	260	220	240	165	283
30	375	345	350	225	398
60	750	700	715	420	800

**Table 5:** Sulfur content of upgraded bitumen at various microwave temperatures and times.

Sulfur Content (wt%)				
	Maximum Microwaving Temperature			
		150 °C	180 °C	200 °C
Microwaving Time	10 min	4.54	4.52	4.51
	20 min	4.50	4.47	4.45

**Table 6:** Physical properties of the bitumen measured at 20 °C.

	Properties	Measured Values	Error Bar (+/−)
Physical Properties	Viscosity (cP)	300,000	1%
	TAN (mgKOH/g)	4.32	0.1
	Density (kg/m^3^)	1020	5.0
	API (°)	7.23	
SARA Fractions	Saturates (wt%)	18.5	0.2
	Aromatics (wt%)	28.9	0.3
	Resins (wt%)	30.6	0.3
	Asphaltenes (wt%)	19.8	0.2

## Data Availability

Data are contained within the article and Supplementary Materials.

## Supplementary Materials

The following supporting information can be downloaded at: https://www.mdpi.com/article/10.3390/molecules28237769/s1.
