# Supplementary material for: Harnessing the Power of Microwave Irradiation: A Novel Approach to Bitumen Partial Upgrading
Source: Molecules. 2023 Nov 25;28(23):7769. doi: 10.3390/molecules28237769 (PMC10707834; doi:10.3390/molecules28237769)
Supplement: Supplementary file 1 [file molecules-28-07769-s001.zip › molecules-2719274-supplementary.pdf]

Supplementary Material

S-1: Detailed SARA procedure:

The Saturates, Aromatics, Resins, and Asphaltenes (SARA) fractions of oil sand bitumen.

I. Separation of Asphaltenes

1) 10±0.5 g bitumen sample is weighted in a pre-weighed 250-mL conical flask, and 100 mL n-pentane is added and is well-mixed with bitumen sample.
2) Warm the liquid mixture on the heat plate for a few seconds with intermittent swirling.
3) Allow the mixture to stand about 30 min at room temperature.
4) Using a 500-mL suction filter to filter the sample. Rinse the conical flask and stirring rod with 60-mL n-pentane, and then pour the rinse through the paper filter.
5) Put the pre-weighted filter paper and residue (asphaltene) in oven overnight in 70°C, after that the weight of residue/asphaltene (W_1) is recorded.
6) Transfer the solution after filtration to a beaker and evaporate the n-pentane in a water bath with the temperature of 45°C on the hot plate.
7) Record the weight of solution (W_2) until the weight loss between the weighs is less than 10 mg and check the weight of Asphaltenes (W_1) with the weight of Asphaltenes by difference (10 - W_2).
8) The solution (W_2) contains the Saturates, Resins, and Aromatics components, and the concentration of each component is determined as following.

II. Separation of Saturates

9) Prepare the Clay-Gel Percolating Column: (a) in the upper section, clay adsorbent of 100 g is added; (b) in the lower section, silica gel of 200 g plus clay of 50 g on top of the silica gel are added.
10) Place a piece of glass wool over the top surface of clay in the upper section to prevent the agitation of the clay while charging the solvents.
11) The solution (W2) is charged with 25 mL n-pentane solvent and is well-mixed to ensure a uniform and stable solution.

12) Charge the Clay-Gel Percolating Column with 25 mL n-pentane and allow to percolate into the clay, and then add the diluted solution to the column.

13) Wash the sample beaker with n-pentane and add the washings to the column.

14) Charge n-pentane to the column and maintain a head level of 20 mm well above the top of the upper column.

15) Collect 280±10 mL of the first n-pentane effluent from the bottom of Clay-Gel Percolating Column in a 500-mL pre-weighed beaker.

16) The n-pentane solvent in 500-mL pre-weighed beaker is evaporated at 45 °C in a water bath, and the weight of Saturates (W3) is obtained by the difference method.

III. Separation of Resins

17) Disconnect the two columns and allow the lower column to drain into a receiver. Continue washing the upper clay section with n-pentane.

18) Maintain a moderate liquid head level above the clay during the washing and adjust n-pentane additions so that the level is about 25 mm when 150 mL have been collected in the receiver, and this n-pentane effluent is part of Aromatics component.

19) Discontinue additions at this point and allow the liquid to essentially drain from the clay column.

20) After n-pentane effluent has essentially drained from the column, charge a 50 to 50 volume mixture of toluene–acetone solution.

21) Collect 250 mL of the toluene-acetone (plus n-pentane) effluent from the bottom of clay column.

22) The solvent toluene-acetone (plus n-pentane) is evaporated at 110°C on a heat plate, and the weight of Resins (W4) is obtained by the difference method.

IV. Separation of Aromatics

1) The gel column (lower section) is placed in the extraction apparatus. Toluene (200±10 mL) is added into a 500-mL flask and refluxed at such a rate of 10 ± 2 mL/min for 2 h.

2) At the end of this time, the valve of extraction apparatus is opened, and the toluene removed into a waste solvent receiver to a volume of approximately 50-mL in the flask. The solution

remaining is then combined with the n-pentane effluent from step 18 for the recovery of aromatics.

3) The solvent toluene is evaporated at 110°C, and the weight of Aromatics (W5) is obtained.

S-2: BET graphs:

1. Activated carbon:

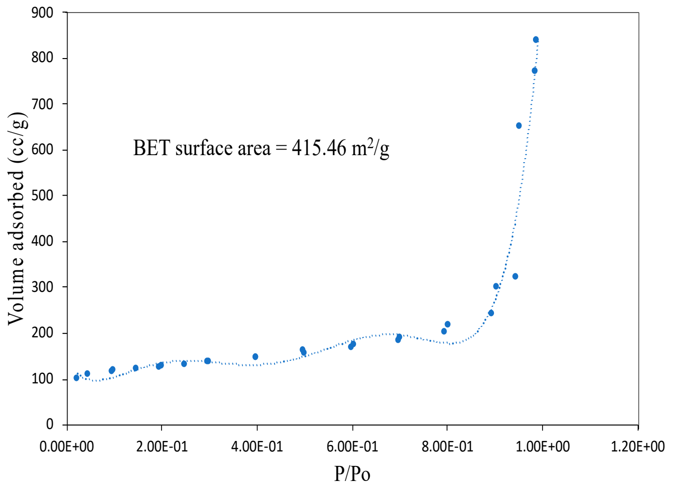

Figure S1. t-Plot Method Micropore Analysis for activated carbon particles

2. Biochar:

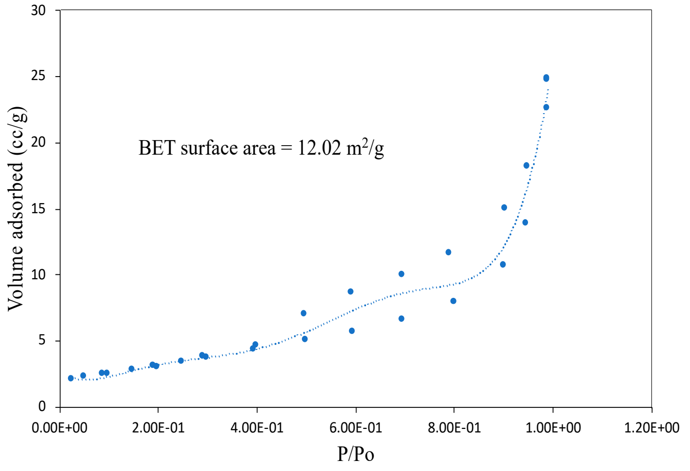

Figure S2. t-Plot Method Micropore Analysis for Biochar particles

3. Coke:

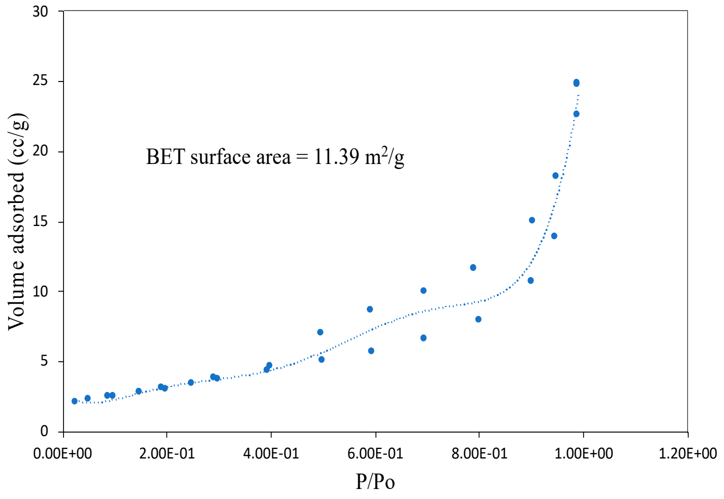

Figure S3. t-Plot Method Micropore Analysis for Coke particles

4. Graphite

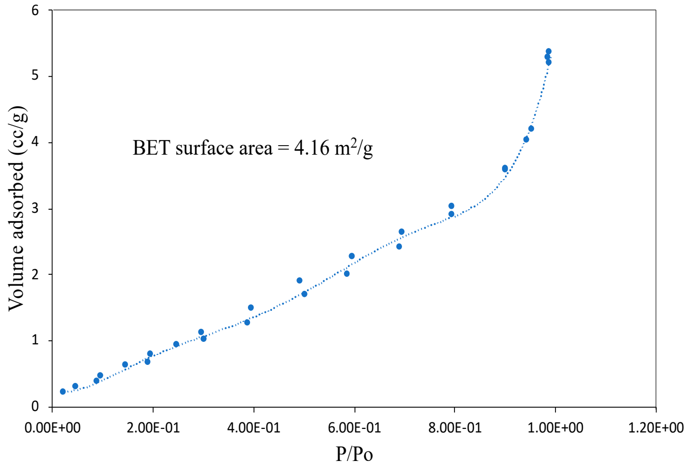

Figure S4. t-Plot Method Micropore Analysis for Graphite particles

5. Activated Biochar

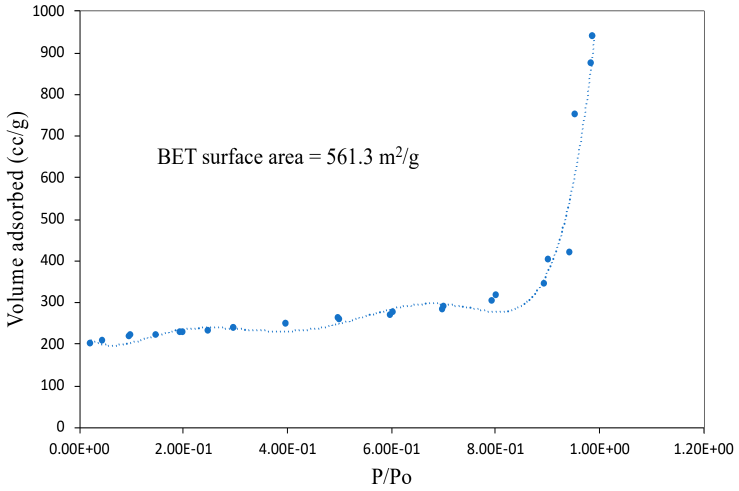

Figure S5. t-Plot Method Micropore Analysis for Activated biochar particles

S-3: Results for leached Activated carbon as a microwave susceptor:

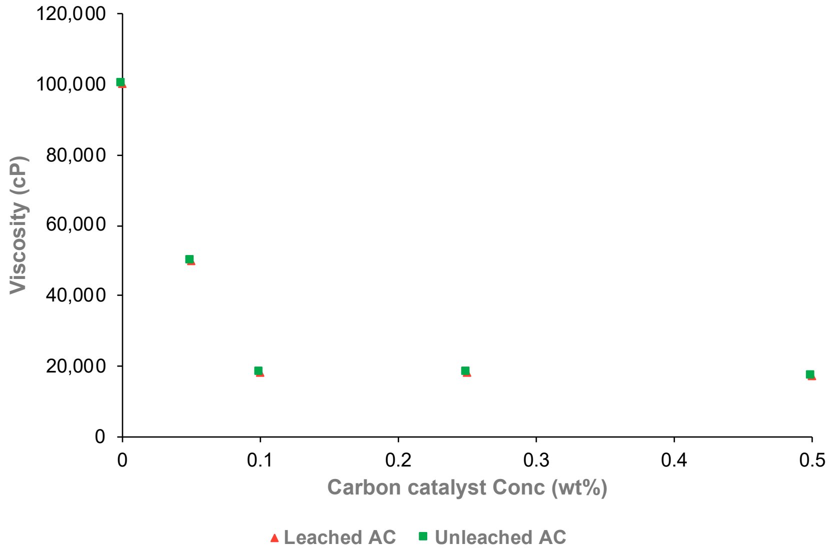

Figure S6. The effect of leached AC on bitumen viscosity reduction at 150°C.

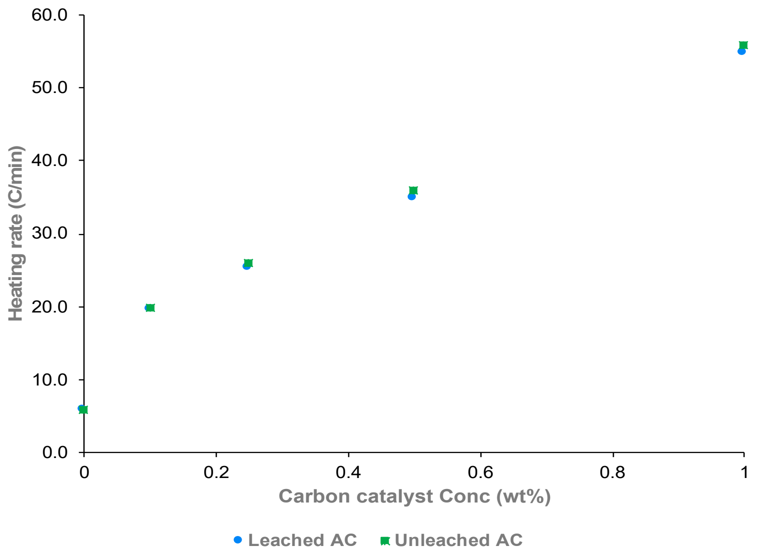

Figure S7. The effect of leached AC on microwave heating rates.
